# The Influence of Blonanserin Supersaturation in Liquid and Silica Stabilised Self-Nanoemulsifying Drug Delivery Systems on In Vitro Solubilisation

**DOI:** 10.3390/pharmaceutics15010284

**Published:** 2023-01-14

**Authors:** Amalie Møller, Hayley B. Schultz, Tahlia R. Meola, Paul Joyce, Anette Müllertz, Clive A. Prestidge

**Affiliations:** 1 UniSA Clinical & Health Sciences, University of South Australia, Adelaide, SA 5000, Australia; 2 Department of Pharmacy, Faculty of Health and Medical Sciences, University of Copenhagen, 2100 Copenhagen, Denmark; 3 Future Industries Institute, UniSA STEM, Mawson Lakes Campus, University of South Australia, Mawson Lakes, SA 5095, Australia; 4 Bioneer:FARMA, Department of Pharmacy, Faculty of Health and Medical Sciences, University of Copenhagen, 2100 Copenhagen, Denmark

**Keywords:** SNEDDS, lipids, supersaturation, porous silica, blonanserin, solubilisation

## Abstract

Reformulating poorly water-soluble drugs as supersaturated lipid-based formulations achieves higher drug loading and potentially improves solubilisation and bioavailability. However, for the weak base blonanserin, silica solidified supersaturated lipid-based formulations have demonstrated reduced in vitro solubilisation compared to their liquid-state counterparts. Therefore, this study aimed to understand the influence of supersaturated drug load on blonanserin solubilisation from liquid and silica solidified supersaturated self-nanoemulsifying drug delivery systems (super-SNEDDS) during in vitro lipolysis. Stable liquid super-SNEDDS with varying drug loads (90–300% of the equilibrium solubility) were solidified by imbibition into porous silica microparticles (1:1 lipid: silica ratio). In vitro lipolysis revealed greater blonanserin solubilisation from liquid super-SNEDDS compared to solid at equivalent drug saturation levels, owing to strong silica-BLON/lipid interactions, evidenced by a significant decrease in blonanserin solubilisation upon addition of silica to a digesting liquid super-SNEDDS. An increase in solid super-SNEDDS drug loading led to increased solubilisation, owing to the increased drug:silica and drug:lipid ratios. Solidifying SNEDDS with silica enables the fabrication of powdered formulations with higher blonanserin loading and greater stability than liquid super-SNEDDS, however at the expense of drug solubilisation. These competing parameters need careful consideration in designing optimal super-SNEDDS for pre-clinical and clinical application.

## 1. Introduction

The prevalence of poorly water-soluble drugs (PWSDs) is ever increasing, as modern drug discovery methodologies such as combinatorial chemistry, high throughput screening and cell-based activity assays tend to identify new chemical entities with large molecular weight and greater lipophilicity [1]. It is estimated that up to 90% of these emerging chemical entities and 75% of compounds in development are poorly water-soluble, which is a major indicator of poor solubility in intestinal fluids, leading to lack of oral absorption, poor bioavailability, and inefficient treatment of patients [2,3,4]. However, the poor oral bioavailability of PWSDs can be circumvented by applying formulation strategies that improve the drug solubility in intestinal fluids, thereby enhancing absorption [5].

One such formulation strategy is to use lipid-based formulations (LBFs) [6]. LBFs bypass the critical absorption rate-limiting dissolution step to enhance the apparent solubility by generating supersaturated drug solutions when exposed to the gastrointestinal environment, as the drug is pre-solubilised within the liquid lipid-based formulation (LBF) when delivered to the gut [7]. The lipid is digested, driving drug release and the formation of solubilising species (mixed micelles) with bile secretions that maintain the drug in solution and promoting drug absorption [8]. The composition of LBF can range from simple mixtures of one lipid and a drug, to more complex systems containing various lipids, surfactants, co-surfactants, co-solvents, and a drug that form emulsions upon dispersion. The various LBF have been classified based on formulation composition by the Lipid Formulation Classification System (LFCS) [9].

Despite their ability to improve the solubilisation and absorption of PWSDs, the commercial success of LBF has been limited, with only six LBF products being commercialised since 2010 [10]. This may be attributed to factors such as costly manufacturing, poor physical and chemical stability, difficult portability, and tendency for drug crystallisation and precipitation in vivo [11]. A viable approach to overcome key limitations associated with LBF is to solidify the liquid-state LBF using a solid carrier to form a solid dosage form. The solidification of lipid formulations retains the beneficial properties associated with the liquid form, whilst imparting additional advantages, including improved drug solubilisation and dissolution, improved safety, controlled drug release, and industrial and commercial benefits [12]. However, when solidifying a liquid-state LBF with a solid carrier, the benefits are offset by a decrease in drug load (% *w*/*w*), restricting the application to low-dose and highly potent drugs.

To mitigate the drug-loading challenges of solid-state LBFs, the PWSD can be loaded within the lipid component at concentrations above its equilibrium solubility (S_eq_) to create a supersaturated formulation [12]. Conventional liquid-state LBFs are loaded below the S_eq_ of the drug in the system (i.e., 75–90% S_eq_); however, supersaturated systems, such as supersaturated SNEDDS (super-SNEDDS), load the drug above the S_eq_ by heating a lipid and drug mixture to dissolve the excess drug, prior to stabilising the thermodynamically unstable system through spatial confinement within the pores of mesoporous silica microparticles as a solid carrier, achieving higher drug loads and a supersaturated solid-state LBF. Schultz et al. investigated the balance between high drug loading through supersaturation, and performance of ibuprofen-loaded supersaturated silica-lipid hybrids (super-SLH) [13,14]. In vivo pharmacokinetic studies in rodents revealed that super-SLH with a supersaturation level of 100% and 227% S_eq_ both achieved a 2.2-fold improvement in bioavailability, compared to the commercially available formulation. However, at a level of 389% S_eq_, the bioavailability was not significantly different from the commercial product. This diminishing effect of super-SLH was attributed to a larger content of crystalline drug and less lipid available for solubilisation, highlighting that high drug loads do not necessarily correlate with improved performance, and a fine balance between drug load and performance exists. Further studies have investigated the application of super-SLH to abiraterone acetate, fenofibrate, simvastatin, and blonanserin (BLON), and have investigated the influence of different types of silica, silica geometry/microporosity, different LFCS LBF types, and liquid- vs. solid-state LBF [15,16,17,18,19,20].

One gap in the literature surrounding super-SLH is the influence of supersaturation level on weak bases such as BLON, and perhaps more significantly, the interaction between BLON and silica that impedes its solubilisation. Previously, Møller et al. investigated the influence of lipid formulation classification type (i.e., type I vs. type II vs. type IIIA/self-nanoemulsifying drug delivery systems (SNEDDS)) and physical state (liquid LBF vs. solidified with silica) on the in vitro solubilisation of BLON from a supersaturated LBF [19]. It was reported that type IIIA formulations and liquid-state supersaturated formulations gave superior in vitro solubilisation, while solidification of the supersaturated LBF with silica significantly impeded drug solubilisation. However, only one supersaturation level was investigated (150% S_eq_), and it is known that the supersaturation level can dictate the in vitro and in vivo performance of super-SLH.

Therefore, this study aimed to assess the influence of supersaturation level on the in vitro solubilisation performance of the weak base BLON from liquid and solid Type IIIA LBF (referred to from herein as SNEDDS) under digesting conditions, to ultimately advance understanding of supersaturated solid-LBF performance to inform advancement of such technologies.

## 2. Materials and Methods

### 2.1. Materials

BLON was obtained from Hangzhou Dayangchem Co., Ltd. (Hangzhou, China). Miglyol 812® (medium chain triglycerides) was obtained from Hamilton Laboratories (Adelaide, Australia), and Capmul® MCM (glyceryl mono- and dicaprylate) was obtained from Abitec (Columbus, OH, USA). Pancreatin lipase, Cremophor EL, and 4-bromophenyl boronic acid (4-BBA) were obtained from Sigma-Aldrich (Castle Hill, Australia). Simulated fasted state intestinal fluid (FaSSIF) powder was obtained from Biorelevant Ltd. (London, UK). Porous silica microparticles (Parteck® SLC 500) (particle size 9–11 µm and pore size 6 nm) were obtained from Merck Millipore (Bayswater, Australia). Sodium hydroxide pellets (NaOH), Trizma® maleate, sodium dihydrogen phosphate (NaH_2_PO_4_), orthophosphate acid, calcium chloride dihydrate (CaCl_2_∙2H_2_O), and sodium chloride (NaCl) were obtained from ChemSupply (Gillman, Australia). HPLC-grade methanol was obtained from a local supplier. High purity Milli Q water (Merck Millipore, Bayswater, Australia) was used throughout the study.

### 2.2. HPLC Methold

BLON was quantified through a high-performance liquid chromatography (HPLC) assay developed by Dening et al. [21]. Determinations were performed using a Shimadzu Prominence Ultra-Fast Liquid Chromatograph (UFLC XR) (Kyoto, Japan) and a Grace LiChrospher RP Select B 5u 250 mm × 4.6 mm column with the oven set to 40 °C. The mobile phase was a combination of methanol and 50 mM NaH2PO4 (pH 3) (75:25, *v*/*v*) at a flow rate of 1.5 mL/min, with effluents analysed by a UV detector at 249 nm. The retention time of BLON was 4.1 min. BLON concentrations were determined via peak area vs. concentration linear calibration curves within the concentration range of 0.025–10 µg/mL (r^2^> 0.99).

### 2.3. Fabrication of Liquid and Solid Super-SNEDDS Formulations

Table 1 displays the liquid and solid super-SNEDDS formulations developed for this study.

#### 2.3.1. Preparation of SNEDDS Preconcentrate

The composition of the SNEDDS preconcentrate used in the current study was based on a previous formulation [19], containing Miglyol 812 (36% *w*/*w*), Capmul MCM (36% *w*/*w*), and Cremophor EL (28% *w*/*w*). The SNEDDS preconcentrate was formulated by rotating the lipid excipients in a glass vial for 1 h.

#### 2.3.2. Preparation of Super-SNEDDS Formulations

Drug loading of the SNEDDS preconcentrate to fabricate liquid super-SNEDDS was performed by weighing the required quantity of BLON and SNEDDS preconcentrate into a 1.5 mL tube, vortex mixing for 30 s, and mixing overnight at 60 °C by rotation to allow for drug solubilisation, prior to cooling to room temperature.

To fabricate solid super-SNEDDS, liquid super-SNEDDS were prepared as above, however prior to the liquid super-SNEDDS cooling to room temperature, the required amount of porous silica microparticles (the solid carrier) was added (1:1 lipid:silica ratio) and mixed using a spatula to facilitate lipid imbibition and produce a dry homogeneous mixture.

#### 2.3.3. Determination of Maximum Supersaturation Level of Liquid Super-SNEDDS

The S_eq_ of BLON in the SNEDDS preconcentrate was determined in a previous study (31.4 ± 1.8 mg/mL) [19]. To determine the maximum BLON supersaturation level, the SNEDDS preconcentrate was loaded at 150%, 200%, 250%, 300%, and 350% of the S_eq_ to fabricate liquid super-SNEDDS using the method described in Section 2.3.2. Supersaturation of the SNEDDS preconcentrate was considered successful if the drug load was dissolved after rotating at 60 °C overnight, with no visual drug crystals appearing upon cooling to room temperature.

### 2.4. Chartacterisation of Liqud Super-SNEDDS

Dynamic light scattering was employed to measure the zeta potential, droplet size, and polydispersity index (PDI) of the resulting emulsions following a 100-fold dilution of the SNEDDS preconcentrate and the liquid super-SNEDDS, using a Malvern Zetasizer Nano ZS (Worcestershire, UK). Measurements were performed in triplicate.

### 2.5. Chartacterisation of Solid Super-SNEDDS

Scanning electron microscopy (SEM) was performed using a Carl Zeiss Merlin microscope with a GEMINI II column (Jena, Germany) to examine the surface morphology of the developed solid formulations. Samples were held in place using double-sided adhesive carbon tape and were sputter-coated with 10 nm platinum prior to imaging at an accelerating voltage of 2 kV.

The crystallinity of BLON within solid super-SNEDDS was examined using differential scanning calorimetry (DSC; TA Instruments Discovery DSC, Rydalmere, Australia) and X-ray powder diffraction (XRPD; Malvern Panalytical Empyrean diffractometer Malvern, UK). Briefly, DSC was performed at Day 1 and 22 (post fabrication) by weighing approximately 2 mg sample into a sealed aluminum pan, prior to heating from 50 to 200 °C at a rate of 10 °C/min, under nitrogen. XRPD was performed on Day 1 post fabrication. Formulations were scanned between 5–40° 2θ at a scanning rate of 2.6 s/point and a step size of 0.02°.

### 2.6. In Vitro Lipid Digestion Studies

In vitro lipolysis experiments were modified based on a previous study [22].

#### 2.6.1. Preparation of Simulated Intestinal Media

FaSSIF media containing a 3-mM bile salt (sodium taurocholate) concentration and 0.75 mM phospholipid (soybean lecithin) concentration was prepared following the manufacturers’ instructions (Biorelevant, London, UK). Then, 2 mM tris maleate, 1.4 mM CaCl_2_∙2H_2_O, and 150 mM NaCl were added to the FaSSIF media according to the Lipid Formulation Classification System Consortium [23,24,25]. Pancreatin extract was prepared as previously described [21,26], by dispersing porcine pancreatin in FaSSIF media without bile salts and phospholipids followed by vortex mixing for 30 s, centrifugation (8500× *g* at 4 °C for 20 min), and collection of the supernatant.

#### 2.6.2. Lipolysis Experimental Procedure

Next, 18 mL of FaSSIF media was continuously stirred at 37 °C in a thermostated glass container. A quantity of formulation equivalent to 3 times the S_eq_ of BLON in undigested FaSSIF (S_eq_ = 53 ± 2 mg/mL) [19] was placed into the container. After dispersion for 1 min, 2 mL pancreatin extract was added to initiate lipolysis. Lipolysis was performed for 60 min, with the pH maintained at 6.5 by auto-titration of 0.06 M NaOH using a pH-stat apparatus (902 Titrando-Metrohm pH-stat and Dosino 800 dosing system). The volume of NaOH used over time was recorded.

A background titration measurement without the addition of a formulation was performed. This background measurement was subtracted from the titration measurement of the formulations to correct for any fatty acids (FA) formed upon hydrolysis of the components in the FaSSIF medium by pancreatin. The volume of NaOH titrated was converted to a molar concentration and directly correlates with the concentration of FA released during lipid digestion. Lipolysis experiments were performed in triplicate.

#### 2.6.3. Drug Partitioning

Aliquots (1 mL) were collected at 1, 5, 10, 15, 30, 45, and 60 min during lipolysis studies to examine the partitioning of BLON between the aqueous and pellet phases. Lipase activity was inhibited by adding 5 µL of 1.0 M 4-BBA (10% *w*/*v* in MeOH) to the collected aliquots. Following this, the samples were centrifugated (10 min at 12,500× *g*) and HPLC was used to determine the BLON concentration in the supernatant following dilution with MeOH. The pellet was dissolved in MeOH via sonication and subsequently separated through centrifugation (10 min at 12,500× *g*), where BLON content within the supernatant was quantified using HPLC.

Lipolysis was also performed to examine BLON solubilisation from liquid super-SNEDDS150% over 90 min with the addition of porous silica microparticles (equivalent to the amount in the solid super-SNEDDS150%) after 30 min.

### 2.7. Lipid Content of Solid Super-SNEDDS Pre- and Post-Lipolysis

The lipid contents of a blank solid super-SNEDDS150%, pre- and post-lipolysis, were measured using thermogravimetric analysis (TGA) using a TA Instruments Discovery TGA (Australia). The digested formulation pellet was isolated and dried following 60 min of lipolysis as described in Section 2.6.3. Approximately 10 mg of sample was weighed into an aluminium pan and heated at a rate of 20 °C/min, from 25 °C to 600 °C, under nitrogen. The lipid decomposed in the temperature range of 150–450 °C, while the silica component remained thermally stable. Accounting for residual water content, the resulting formulation weight loss corresponded approximately to the lipid content of the samples.

### 2.8. ^1^H NMR of Lipid Digestion Products

The lipid species in the solid super-SNEDDS150% pre- and post-lipolysis were determined by coupling proton nuclear magnetic resonance (^1^H NMR) spectroscopy. The digested formulation pellet was isolated and dried following 60 min of lipolysis as described in Section 2.6.3. The solid samples were prepared for 1H NMR as previously described [27], extracting lipid by dispersing the sample in 1 mL dichloromethane, followed by centrifugation at 29,000× *g* for 5 min. The dichloromethane phase was evaporated under vacuum at room temperature, resulting in lipid films that were dissolved in 1 mL deuterated chloroform for ^1^H NMR analysis. ^1^H NMR spectra was obtained using a Bruker Ultrashield 500 (Billerica, USA) with an acquisition time of 4.819 s and pulse width of 90°. Lipid species, including triglycerides (TG), diglycerides (DG), monoglycerides (MG), FA, and monoesters, were quantified as previously described [28,29,30].

## 3. Results and Discussion

### 3.1. Development of Super-SNEDDS for BLON

The SNEDDS preconcentrate investigated in this study was composed of 36% (*w*/*w*) Miglyol 812, 36% (*w*/*w*) Capmul MCM and 28% (*w*/*w*) Cremophor EL. The BLON S_eq_ of the SNEDDS preconcentrate was 31.4 ± 1.8 mg/mL at room temperature. The SNEDDS preconcentrate was able to supersaturate BLON as high as 300% S_eq_; however, the formulation was prone to precipitation unless stabilised through the addition of silica to form a solid powder. Therefore, the greatest supersaturation level selected for investigation was 250% S_eq_ in liquid super-SNEDDS and 300% S_eq_ in solid super-SNEDDS. The formulations are summarised in Table 1. All formulations were used within 24 h of preparation if not otherwise specified.

### 3.2. Characterisation of Super-SNEDDS

#### 3.2.1. Liquid Super-SNEDDS

The particle size, polydispersity, and zeta potential characteristics of blank SNEDDS preconcentrate and liquid super-SNEDDS are presented in Table 2. The droplet size of blank SNEDDS preconcentrate was measured to be 39.2 nm with a PDI of 0.029. Following BLON loading, the droplet size decreased by 6.2–8.3 nm, whereas the PDI increased by 0.087–0.104. The increase in PDI could be due to BLON being added to the system and not contributing to emulsification, hence disturbing the synergy between lipids, surfactant, and media [31]. Previously, a decrease in droplet size was also observed upon loading fenofibrate into SNEDDS, where the authors attributed the decrease to fenofibrate having both a hydrophobic and hydrophilic domain that could provide surface active properties [32]. The blank SNEDDS preconcentrate measured a zeta potential of −1.69 mV. The zeta potential increased by 8.77–11.6 mV when BLON was loaded into the SNEDDS due to BLON being positively charged. These small droplet sizes and positive zeta potentials are ideal characteristics for effective SNEDDS drug delivery.

The two liquid super-SNEDDS were stored for 14 days at room temperature and observed for crystalline BLON to determine their stability. Crystalline BLON was observed in liquid super-SNEDDS250% after 14 days of storage; however, no crystalline BLON was observed in liquid super-SNEDDS150% after the 14 days.

#### 3.2.2. Solid Super-SNEDDS

The SEM images illustrating the surface morphology of blank porous silica microparticles and solid super-SNEDDS are displayed in Figure S1. The blank silica microparticles were irregular in shape with a diameter ranging from 7 to 11 µm. Regardless of the BLON supersaturation level, the solid super-SNEDDS particles did not appear any different to the blank porous silica microparticles, which suggests complete drug loading was achieved. Previous studies have demonstrated that SEM can visualise precipitated drug and/or lipid expelled from silica pores [14]; however, this was not apparent in the present study.

**Figure 1:**
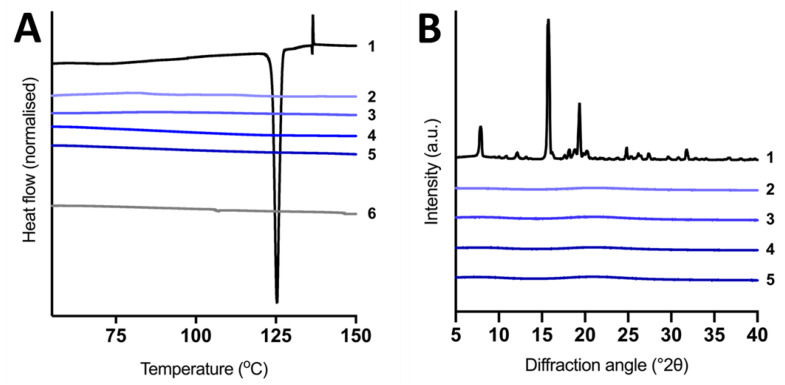
(**A**) DSC thermograms (endothermic down) and (**B**) XRPD patterns. 1—Crystalline BLON, 2—solid super-SNEDDS90%, 3—solid super-SNEDDS150%, 4—solid super-SNEDDS200%, 5—solid super-SNEDDS250% and 6—blank silica microparticles. Data for crystalline BLON, solid super-SNEDDS150%, and blank silica microparticles was previously reported by and is published with permission from [19].

The DSC thermograms of BLON and solid super-SNEDDS are displayed in Figure 1A. No endothermic or exothermic peaks were observed in the thermogram for blank silica microparticles as silica does not undergo any thermal transitions. In comparison, crystalline BLON melted and displayed an endothermic peak at 124 °C, corresponding to its melting point [33]. At the BLON melting point, an endothermic peak was observed for the 1% *w*/*w* physical mixture of BLON in silica microparticles (Figure S2), validating that crystalline BLON could be detected as low as this concentration using this DSC method. No exothermic or endothermic peaks were detected for any of the solid super-SNEDDS after 1 day or 22 days following fabrication (Figure S2), confirming negligible precipitation of supersaturated BLON over this time period.

The XRPD patterns of BLON and solid super-SNEDDS at varying supersaturation levels are displayed in Figure 1B. Crystalline BLON exhibited intense characteristic peaks at 7.8, 15.7, and 19.4°, which were in agreement with the literature [21,26]. A 1% *w*/*w* and 5% *w*/*w* physical mixture of BLON in silica microparticles displayed the same characteristic peaks for BLON (Figure S3), indicating that a concentration as low as 1% crystalline BLON could be detected using this method. All the XRPD patterns for the solid super-SNEDDS, regardless of the supersaturation level, contained no peaks, indicating the solid super-SNEDDS maintain the supersaturated BLON in the non-crystalline state after fabrication (1 day).

The evidence attained from SEM imaging, DSC, and XRPD analysis suggest that the liquid super-SNEDDS were adsorbed within the pores of the porous silica, which maintained BLON in its non-crystalline state at supersaturation levels as high as 300% S_eq_. The silica surface and small pore size (6 nm) may aid in the stabilisation of the supersaturated BLON, thus restricting/inhibiting drug precipitation [14,34,35].

### 3.3. BLON Solubilisation during In Vitro Lipolysis of Super-SNEDDS

The S_eq_ of BLON in FaSSIF was 53 ± 1.6 µL/mL. The super-SNEDDS were dosed at an equivalent of 2.7 mg of BLON to the lipolysis container, achieving non-sink conditions at 3 times S_eq_, but resulting in different amounts of lipid and silica dosed for each formulation (Table S1).

#### 3.3.1. Influence of BLON Load on Solubilisation Performance of Liquid Super-SNEDDS

**Figure 2:**
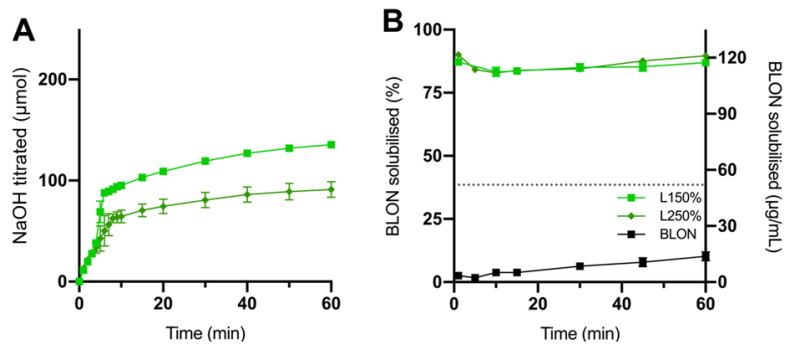
The (**A**) digestion profiles represented as NaOH titrated and (**B**) BLON solubilisation profiles during the in vitro lipolysis (pH 6.5) of liquid super-SNEDDS150% (light green square, L150%), liquid super-SNEDDS250% (dark green diamond, L250%), and pure BLON (black square). BLON S_eq_ in undigested media is represented by the dotted line. Values represent the mean ± SD, *n* = 3. Data for crystalline BLON and liquid super-SNEDDS150% was previously reported by and is published with permission from [19].

The digestion and BLON solubilisation profiles observed for liquid super-SNEDDS150% and liquid super-SNEDDS250% are displayed in Figure 2. The observed increase in the amount of NaOH titrated throughout lipolysis is directly correlated with the generation of FA. The liquid super-SNEDDS150% achieved approximately 50% greater extent of lipolysis after 60 min compared to liquid super-SNEDDS250% (Figure 2A), owing to the higher lipid dose (55 mg vs. 35 mg). However, upon adjusting digestion for the amount of lipid dosed, the extent of lipolysis for both liquid super-SNEDDS were similar (Figure S4). Both liquid super-SNEDDS (liquid super-SNEDDS150% and liquid super-SNEDDS250%) initially displayed rapid digestion over 7 min followed by a slow and continuous digestion until the end of the experiment.

Pure BLON displayed poor solubilisation during lipolysis, reaching only 10% solubilisation after 60 min, owing to BLON being crystalline, possessing a low surface area to volume ratio and a high logP value (logP = 5.5) [33]. In contrast, both liquid super-SNEDDS maintained approximately 85% BLON dose solubilisation throughout lipolysis (Figure 2B) and were able to maintain greater concentrations of BLON than the S_eq_ in FaSSIF solubilised during the lipolysis, thus exhibiting considerably greater BLON solubilisation than pure BLON over the 60 min (AUC_0–60_ >13-fold).

In a previous study by Thomas et al. [36], liquid super-SNEDDS containing simvastatin at drug loading levels of 150% and 200% S_eq_ were investigated. Both formulations solubilised a greater concentration of simvastatin than the S_eq_ of the lipolysis media, with the 200% loaded super-SNEDDS displaying greater solubilisation during the initial 45 min of lipolysis compared to the 150% loaded super-SNEDDS. However, the 200% super-SNEDDS exhibited a rapid precipitation after 30 min of lipolysis. In the current study, no drug precipitation occurred for either liquid super-SNEDDS during lipolysis, however crystalline BLON was observed in liquid super-SNEDDS250% after 14 days of storage and not in liquid super-SNEDDS150%. Thus, liquid super-SNEDDS150% was considered the optimal of the two liquid formulations due to their equivalent in vitro performance and greater stability.

#### 3.3.2. Influence of BLON Load on Solubilisation Performance of Solid Super-SNEDDS

**Figure 3:**
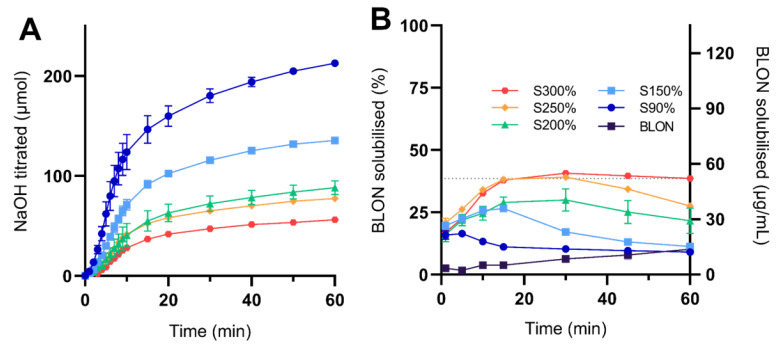
The (**A**) digestion profiles represented as NaOH titrated and (**B**) BLON solubilisation profiles during in vitro lipolysis (pH 6.5) of solid super-SNEDDS300% (red hexagon), solid super-SNEDDS250% (orange diamond, S250%), solid super-SNEDDS200% (green triangle, S200%), solid super-SNEDDS150% (light blue square, S150%), solid super-SNEDDS90% (blue circle, S90%), and pure BLON (black square), all dosed at the same BLON dose. BLON S_eq_ in FaSSIF is represented by the dotted line. Values represent the mean ± SD, *n* = 3. Data for crystalline BLON and solid super-SNEDDS150% was previously reported by and is published with permission from [19].

The digestion and BLON solubilisation profiles observed from solid super-SNEDDS, at drug loading levels of 90%, 150%, 200%, 250%, and 300%, are displayed in Figure 3.

With an increase in drug load there was a decrease in digestion. As with the liquid super-SNEDDS, supersaturation levels within solid super-SNEDDS were inversely proportional to lipid dose (Table S1), hence there is less lipid available for digestion (ranging from 26 mg to 93 mg). After adjusting the digestion for the amount of lipid dosed (Figure S4), all solid super-SNEDDS displayed a similar rate and extent of digestion.

During in vitro lipolysis, the BLON solubilisation profiles for all solid super-SNEDDS displayed an increase followed by a decrease. The greater the drug loading, the later the solubilisation peak occurred and the greater the BLON solubilisation during the 60 min lipolysis period (AUC0–60, Figure S5). All solid super-SNEDDS solubilised BLON at a lower concentration than the S_eq_ in FaSSIF. However, all solid super-SNEDDS exhibited considerably greater BLON solubilisation than pure BLON over 60 min (AUC0–60 ranging from 1.8- to 6.0-fold), yet lower BLON solubilisation than the liquid super-SNEDDS. A strong positive correlation existed between the supersaturation level and the BLON solubilisation (AUC0–60) (r^2^ = 0.97). However, the difference in BLON solubilisation between solid super-SNEDDS250% and solid super-SNEDDS300% was less than between solid super-SNEDDS200% and solid super-SNEDDS250%, suggesting that the benefit of increasing supersaturation level to increase BLON solubilisation approaches a plateau.

A previous study demonstrated that abiraterone acetate loaded super-SLH formulations containing supersaturated drug loads of 150%, 200%, and 250% S_eq_ produced similar drug solubilisation profiles during lipolysis despite their different drug loads [15]. In contrast, another study demonstrated that ibuprofen-loaded super-SLH formulations containing supersaturated drug loads of 100%, 227%, and 389% S_eq_ displayed a reduction in ibuprofen solubilisation with an increase in drug load during dissolution studies [14]. Furthermore, in the present study, an increase in supersaturated drug load resulted in an increase in BLON solubilisation during lipolysis. Therefore, it is suggested that the relationship between level of drug supersaturation and drug solubilisation performance of silica solidified supersaturated LBF is drug-dependent.

#### 3.3.3. The Influence of Silica on BLON Solubilisation

**Figure 4:**
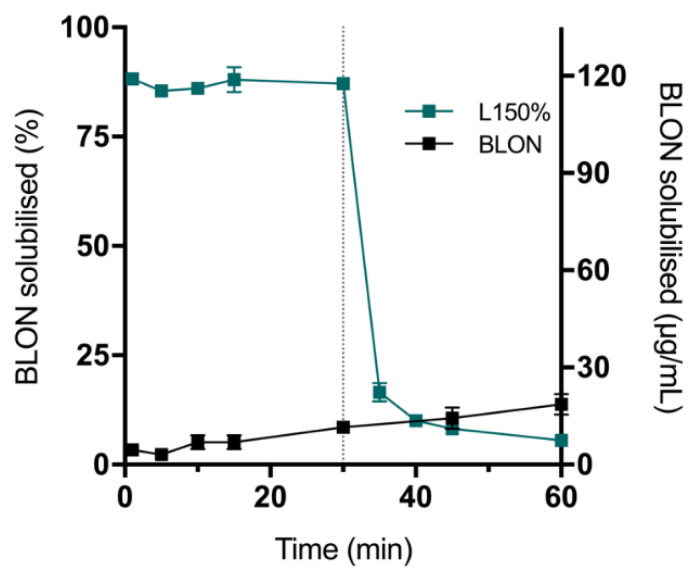
BLON solubilisation profiles during in vitro lipolysis (pH 6.5) of liquid super-SNEDDS150% (teal square, L150%) with silica added after 30 min (grey dotted line) and crystalline BLON (black square). Values represent the mean ± SD, *n* = 3. Data for crystalline BLON was previously reported by and is published with permission from [19].

In attempt to explain why solid super-SNEDDS performed relatively poorly compared to liquid super-SNEDDS, lipolysis of liquid super-SNEDDS150% was performed with the addition of silica microparticles at 30 min (Figure 4, Figures S6 and S7). The addition of silica appeared to have no influence on the digestion profile of liquid super-SNEDDS150%. However, it had a substantial effect on BLON solubilisation, decreasing BLON solubilisation from approximately 85% to 17% within 5 min, and continuing to decrease BLON solubilisation reaching 4.1% at 60 min (less than pure BLON). This may be explained by the physicochemical characteristics of BLON and silica. BLON has a pKa of 7.7 [33] and is therefore protonated in pH 6.5 FaSSIF media, whereas silica is negatively charged [27,28,37]. Consequently, it is sensible to propose that the negatively charged silica interacts with positively charged BLON, drawing BLON out of solution. An equivalent phenomenon has been described between the weak base risperidone and silica [38]. The interaction could also explain the rise and fall in the solubilisation of BLON that was observed from the solubilisation profiles of the solid super-SNEDDS; as lipid digestion and/or the presence of the media drives the release of the BLON-loaded lipid from the silica, the BLON is solubilised in the media and micelles, and is subsequently readsorbed by the silica.

### 3.4. Lipid Release and Digestion Products of Solid Super-SNEDDS

**Figure 5:**
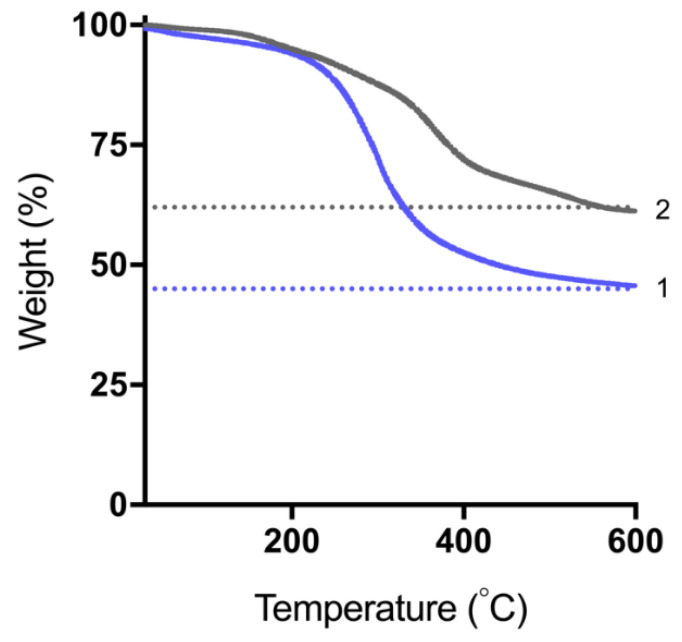
TGA thermograms of a blank solid super-SNEDDS150% 1—before and 2—after in vitro digestion, over the temperature range 25 °C to 600 °C. Horizontal dotted lines indicate the final weight of each sample.

The lipid content of a blank solid super-SNEDDS150% pre- and post-lipolysis was quantified using TGA to determine what percentage of lipid was released during lipolysis (Figure 5), where post-lipolysis was the isolated dried pellet after 60 min of lipolysis of the formulation. Water loss accounted for approximately 2–5% of the weight of the formulations, while BLON decomposition was considered relatively negligible. These lipids, but not silica, are known to decompose at the examined temperature range. Lipid decomposition accounted for 50% of the formulation pre-lipolysis, which agreed with the loaded lipid content of 50% *w*/*w*, and 36% of the formulation post-lipolysis, indicating that more than half of the loaded lipid was retained in the silica during 60 min of lipolysis. This phenomenon has been previously described in the literature, whereby lipid was retained in the pores of silica hindering drug release [39]. Owing to its high lipophilicity, BLON favours the lipophilic environment, rather than partitioning into the aqueous media, thus when lipid is retained in the silica, BLON is also retained. Furthermore, due to the suspected BLON-silica interactions, a portion of free BLON is expected to be retained within silica due to electrostatic interactions and in a solubilised form due to confinement within pores (even without lipid being present).

**Figure 6:**
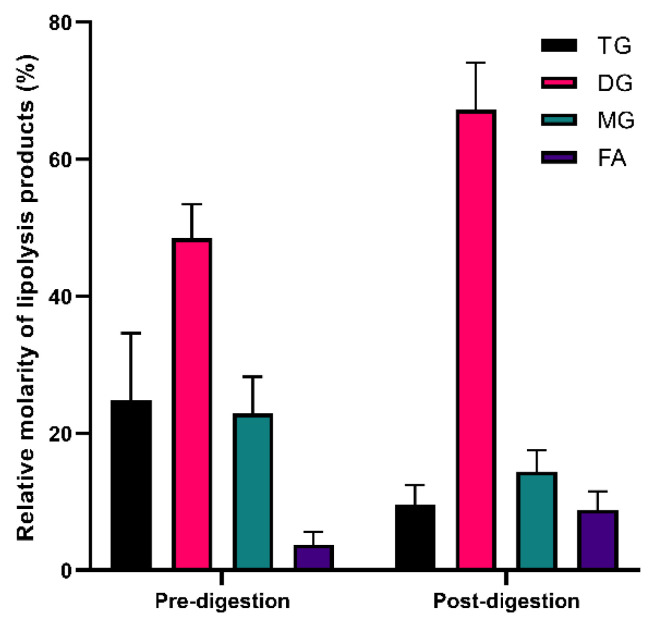
Relative lipolytic product adsorption and speciation within the undigested solid super-SNEDDS150% and after 60 min of lipolysis (pH 6.5) of solid super-SNEDDS150%. Values represent the mean ± SD, *n* = 3. Abbreviations: TG, triglyceride; DG, diglyceride; MG, monoglyceride; FA, fatty acid.

To further understand the lipolysis characteristics of the solid super-SNEDDS, NMR was used to quantify the relative lipolytic product adsorption for solid super-SNEDDS150% pre- and post-digestion (the lipolysis pellet) (Figure 6). The ^1^H NMR spectra of lipid species present in the solid super-SNEDDS pre- and post-digestion are provided in Figure S8. It was assumed that the dried pellet after 60 min of lipolysis contained lipid associated with silica. Pre-digestion, the lipid content of solid super-SNEDDS contained 25% TG, 48% DG, 23% MG, and 4% FA, whereas post-digestion it contained 10% TG, 67% DG, 14% MG, and 9% FA. As expected, the proportion of TG decreased after undergoing digestion. Post-digestion, the major lipolytic product present was DG, with relatively little MG and FA. This is in accordance with published data which demonstrated that TG and DG adsorb more strongly to silica than MG and FA; repulsive electrostatic interactions between the negatively charged silica, MG, and FA triggers the repulsion of MG and FA from the silica pores [28]. This likely limits the solubilization capacity of mixed micelles within the aqueous phase and prevented BLON from partitioning out of the porous silica, thus limiting aqueous BLON solubilisation from solid super-SNEDDS.

### 3.5. Liquid vs. Solid Super-SNEDDS and Influence of Supersaturation Level

Formulating pure BLON as a liquid or solid super-SNEDDS improved BLON solubilisation considerably. Liquid super-SNEDDS increased BLON solubilisation by 13.4- and 14.2-fold at drug loads of 150% and 250%, respectively, whereas solid super-SNEDDS increased solubilisation by 1.8-, 3.0-, 4.3-, 5.6-, and 6.0-fold at drug loads of 90%, 150%, 200%, 250%, and 300%, respectively. These trends were observed owing to the silica: BLON electrostatic interactions that occurred, whereby the silica extracted BLON out of solution and reduced solubilisation. The increase in in vitro BLON solubilisation during lipolysis with increasing supersaturation levels in solid super-SNEDDS can be attributed to the increases in BLON: silica and BLON:lipid ratios.

Liquid super-SNEDDS clearly demonstrated superior BLON solubilisation over solid super-SNEDDS, attributed to the strong attraction between positively charged BLON and negatively charged silica, as well as the retention of SNEDDS within the silica pores. However, all solid super-SNEDDS demonstrated greater stability than liquid super-SNEDDS, i.e., no visual change in physicochemical characteristics after 21 days of storage, compared to liquid super-SNEDDS250%, which showed visual signs of BLON precipitation after 12 h, and liquid super-SNEDDS150% which showed no visual signs of precipitation after 14 days. It is common for liquid supersaturated formulations to possess limited stability [12]. The pore size, 6 nm, likely contributes to the solubilisation and stability characteristics of the solid super-SNEDDS, and a smaller or larger pore size has the potential to optimise the performance. Furthermore, a solid formulation such as solid super-SNEDDS possess additional benefits such as controlled drug release, ease of handling, and commercial advantages [12] that are not possible for liquid formulations. The intended use for a super-SNEDDS formulation containing a weak base, such as BLON, should be considered when advancing preclinical or clinical development of either a liquid or solid formulation. Regardless of the super-SNEDDS being liquid or solid, a higher supersaturated drug load resulted in greater BLON solubilisation, which according to the available literature, is drug dependent. Care must be taken when selecting the appropriate solid excipient and pore size to form a solid super-SNEDDS, as the interactions between the solid carrier, lipid, and drug must be controlled to ensure the drug partitions to the aqueous phase within the solubilising nanoemulsion.

## 4. Conclusions

Liquid and silica solidified super-SNEDDS containing BLON were successfully formulated at various supersaturated drug loads and the influence of supersaturation level on in vitro BLON solubilisation during in vitro lipolysis quantified. All super-SNEDDS enhanced the solubilisation of pure BLON. An increase in supersaturation level led to increased BLON solubilisation for both liquid and solid super-SNEDDS over the drug load range of 90 to 300% S_eq_. The interactions between negatively charged silica and positively charged BLON resulted in the liquid super-SNEDDS achieving greater BLON solubilisation than the solid super-SNEDDS. When deciding on a supersaturated LBF to deliver a specific drug, the physicochemical properties of the drug and the intended use of the formulation need to be taken into consideration, as both liquid and silica solidified super-SNEDDS present different properties and benefits to the formulation scientist. The findings of this research contribute to the existing knowledge of supersaturated LBF to enhance PWSD delivery.

## Figures and Tables

**Table 1:** Loading level of BLON and formulation compositions of the investigated SNEDDS.

Formulation	Drug Load (% of S_eq_)	Formulation Composition		
		BLON (% *w*/*w*)	SNEDDS Preconcentrate (% *w*/*w*)	Silica (% *w*/*w*)
Liquid super-SNEDDS150%	150	4.7	95.3	0
Liquid super-SNEDDS250%	250	7.6	92.4	0
Solid SNEDDS90%	90	1.4	48.6	50
Solid super-SNEDDS150%	150	2.4	47.6	50
Solid super-SNEDDS200%	200	3.1	46.9	50
Solid super-SNEDDS250%	250	3.9	46.1	50
Solid super-SNEDDS300%	300	4.7	45.3	50

**Table 2:** Droplet size, poly dispersity index (PDI), and zeta potential of liquid SNEDDS dispersed in Milli Q water (diluted 100-fold). Values represent the mean ± SD, *n* = 3.

Formulation	Droplet Size (nm)	PDI	Zeta Potential (mV)
Blank SNEDDS preconcentrate ^1^	39.2 ± 0.4	0.029 ± 0.008	−1.69 ± 0.2
Liquid SNEDDS90%	30.9 ± 0.3	0.116 ± 0.007	8.77 ± 0.6
Liquid super-SNEDDS150% ^1^	31.1 ± 0.3	0.127 ± 0.004	10.1 ± 0.8
Liquid super-SNEDDS200%	31.9 ± 0.3	0.125 ± 0.003	11.6 ± 0.76
Liquid super-SNEDDS250%	33.0 ± 0.3	0.133 ± 0.001	9.81 ± 0.6

^1^ Literature values from [19].

## Data Availability

Data available upon request.

## Supplementary Materials

Supporting information can be downloaded at: https://www.mdpi.com/article/10.3390/pharmaceutics15010284/s1, Figure S1: SEM images of various formulations; Figure S2: DSC thermograms of solid formulations after storage; Figure S3: XRPD patterns of physical mixtures; Figure S4: Digestion profiles corrected for lipid amount; Figure S5: In vitro AUC for formulations; Figure S6: BLON distribution between phases; Figure S7: Digestion profiles; Figure S8: NMR spectra; Table S1: Formulation information.
