# Supplementary material for: The Influence of Blonanserin Supersaturation in Liquid and Silica Stabilised Self-Nanoemulsifying Drug Delivery Systems on In Vitro Solubilisation
Source: Pharmaceutics. 2023 Jan 14;15(1):284. doi: 10.3390/pharmaceutics15010284 (PMC9864080; doi:10.3390/pharmaceutics15010284)
Supplement: Supplementary file 1 [file pharmaceutics-15-00284-s001.zip › pharmaceutics-2082262-supplementary.pdf]

The Influence of Blonanserin Supersaturation in Liquid and Silica Stabilised Self-Nanoemulsifying Drug Delivery Systems on In Vitro Solubilisation

Amalie Møller [a,b,c,$] & Hayley B. Schultz [a,$], Tahlia R. Meola [a], Paul Joyce [a], Anette Müllertz[b,d] & Clive A. Prestidge [a,]*

[a] UniSA Clinical & Health Sciences, University of South Australia, Adelaide, South Australia 5000, Australia

[b] Department of Pharmacy, Faculty of Health and Medical Sciences, University of Copenhagen, 2100 Copenhagen, Denmark

[c] Future Industries Institute, UniSA STEM, University of South Australia, Mawson Lakes Campus, Mawson Lakes, South Australia 5095, Australia

[d] Bioneer:FARMA, Department of Pharmacy, Faculty of Health and Medical Sciences, University of Copenhagen, 2100 Copenhagen, Denmark

[$] Equal first authors

*Corresponding Author – Email: clive.prestidge@unisa.edu.au. Phone: +61 8 830 22438. Postal address: UniSA Clinical & Health Sciences, University of South Australia, Adelaide, South Australia 5000, Australia

SUPPLEMENTARY DATA

Table S1: The drug loading level of BLON, and the amount dose of BLON, lipid and silica of the developed super-SNEDDS for lipolysis.

**Formulation**	**Drug load**	**Amount dosed**		
	**(% of S_{eq})**	**BLON (mg)**	**Lipid (mg)**	**Silica (mg)**
Liquid super-SNEDDS150%	150	2.7	55	0
Liquid super-SNEDDS250%	250	2.7	35	0
Solid SNEDDS90%	90	2.7	91.3	96
Solid super-SNEDDS150%	150	2.7	52.3	57
Solid super-SNEDDS200%	200	2.7	37.3	43
Solid super-SNEDDS250%	250	2.7	29.3	34
Solid super-SNEDDS300%	300	2.7	23.3	29

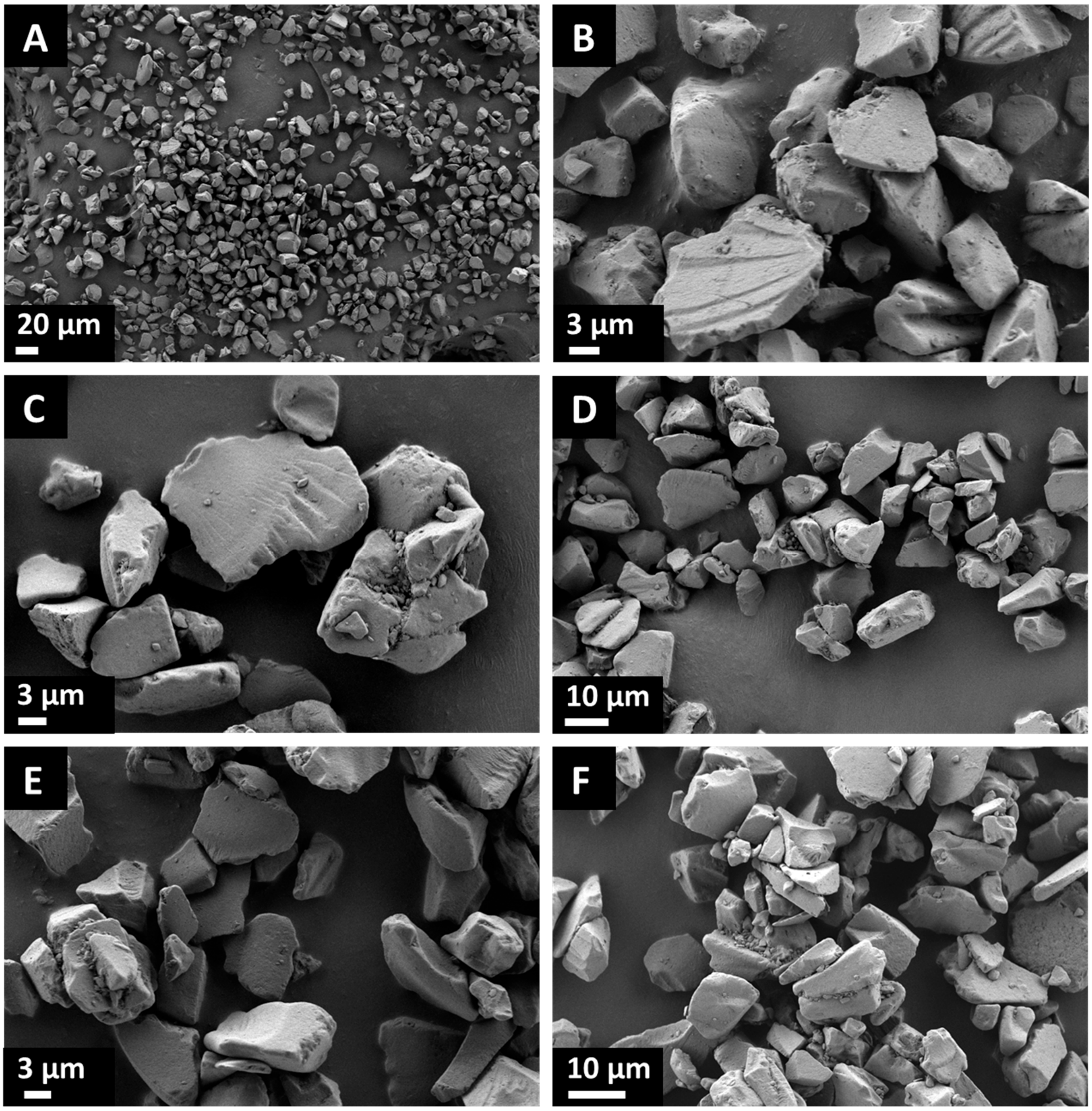

Figure S1: SEM images of silica carrier and solid super-SNEDDS at different drug loading levels. A) and B) mesoporous silica carrier, C) solid super-SNEDDS90%, D) solid super-SNEDDS150%, E) solid super-SNEDDS200% and F) solid super-SNEDDS250%. Data for blank mesoporous silica (A and B) and solid super-SNEDDS150% (D) was previously reported by and is published with permission from (Møller et al., 2021).

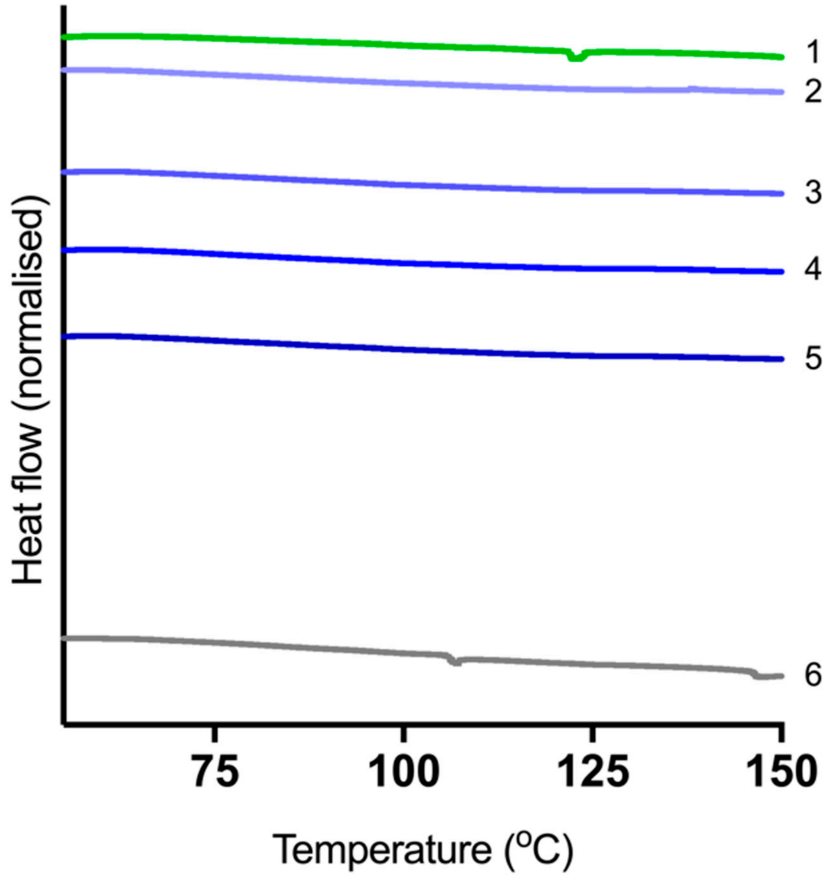

Figure S2: DSC thermograms of solid formulations after 22 days of storage. Endothermic down. 1) 1% physical mixture (w/w) of BLON in silica microparticles, 2) solid super-SNEDDS90%, 3) solid super-SNEDDS150%, 4) solid super-SNEDDS200%, 5) solid super-SNEDDS250% and 6) blank silica microparticles. Data for 1% physical mixture (w/w) of BLON in silica microparticles, solid super-SNEDDS150% and blank silica microparticles was previously reported by and is published with permission from (Møller et al., 2021).

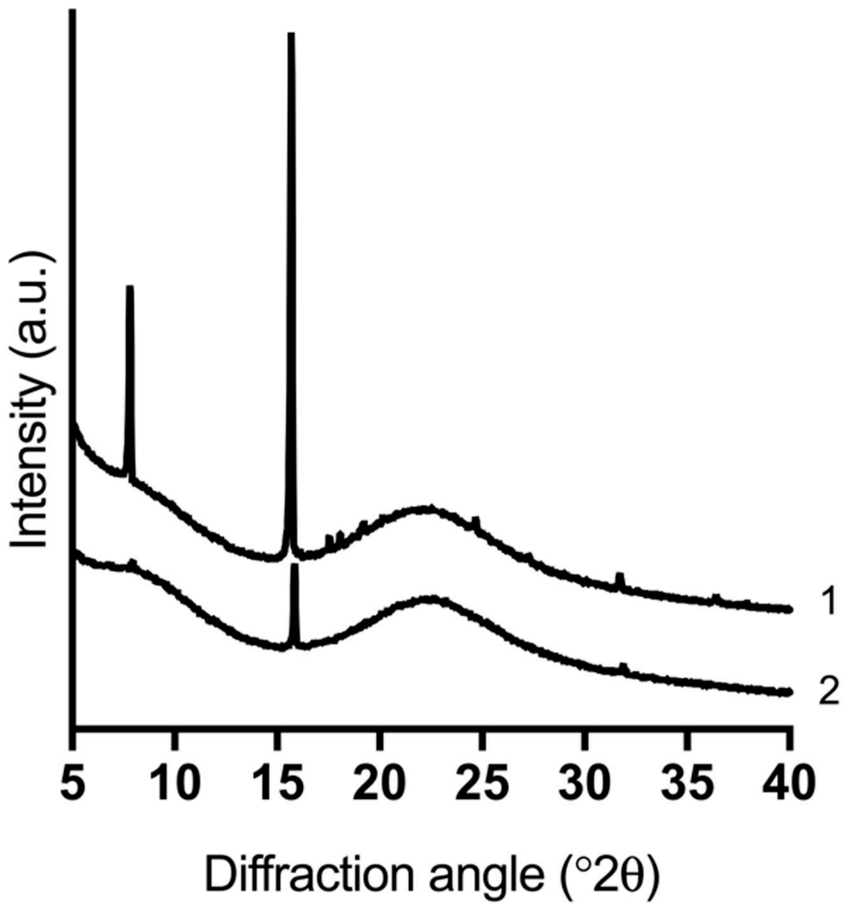

Figure S3: XRPD patterns for 1) 5% and 2) 1% physical mixture (w/w) of BLON in silica microparticles. Data for 5% and 1% physical mixture (w/w) of BLON in silica microparticles was previously reported by and is published with permission from (Møller et al., 2021).

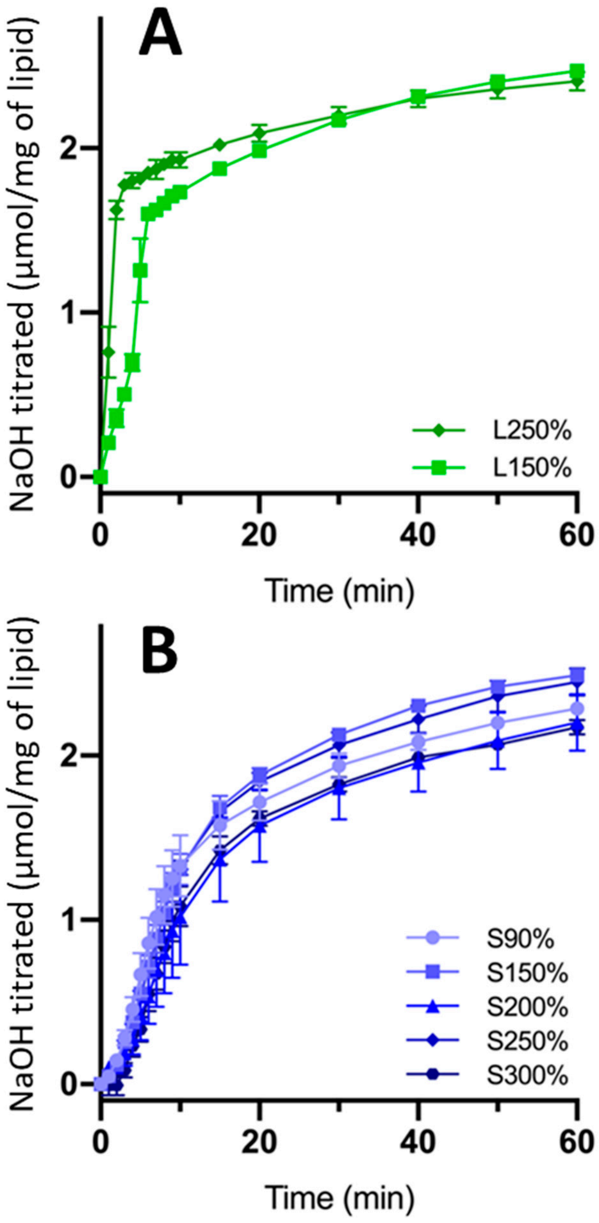

Figure S4: The digestion profiles (corrected for lipid amount in vessel) during *in vitro* lipolysis (pH 6.5) of liquid super-SNEDDS150% (square, light green, L150%), liquid super-SNEDDS250% (diamond, dark green, L250%), solid super-SNEDDS90%, 150%, 200%, 250% and 300% (blue, darker with increasing drug load, circle S90%, square S150%, triangle S200%, diamond S250% and octagon S300%). Each value represents the mean ± SD, n = 3. Data for crystalline BLON, liquid super-SNEDDS150% and solid

super-SNEDDS150% was previously reported by and is published with permission from (Møller et al., 2021).

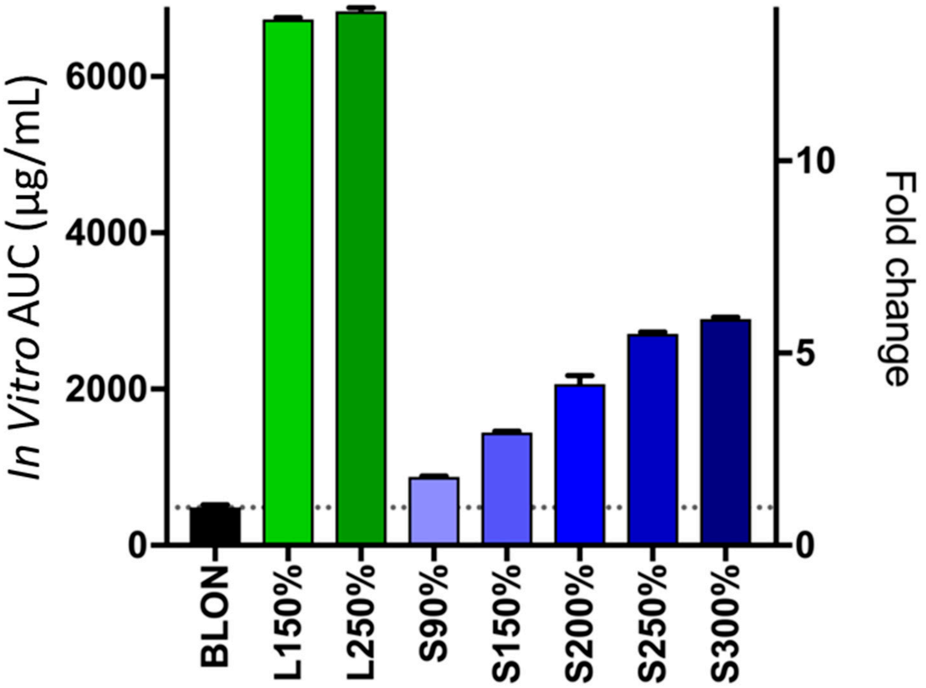

Figure S5: *In vitro* AUC (0-60 min) for BLON solubilisation from different formulations during lipolysis. BLON (black), liquid super-SNEDDS150% (light green), liquid super-SNEDDS250% (dark green) and solid super-SNEDDS90%, 150%, 200%, 250% and 300% (blue, darker with increasing drug load). Grey dotted line indicates AUC for pure BLON. Each value represents the mean ± SD, n = 3. Data for crystalline BLON, liquid super-SNEDDS150% and solid super-SNEDDS150% was previously reported by and is published with permission from (Møller et al., 2021).

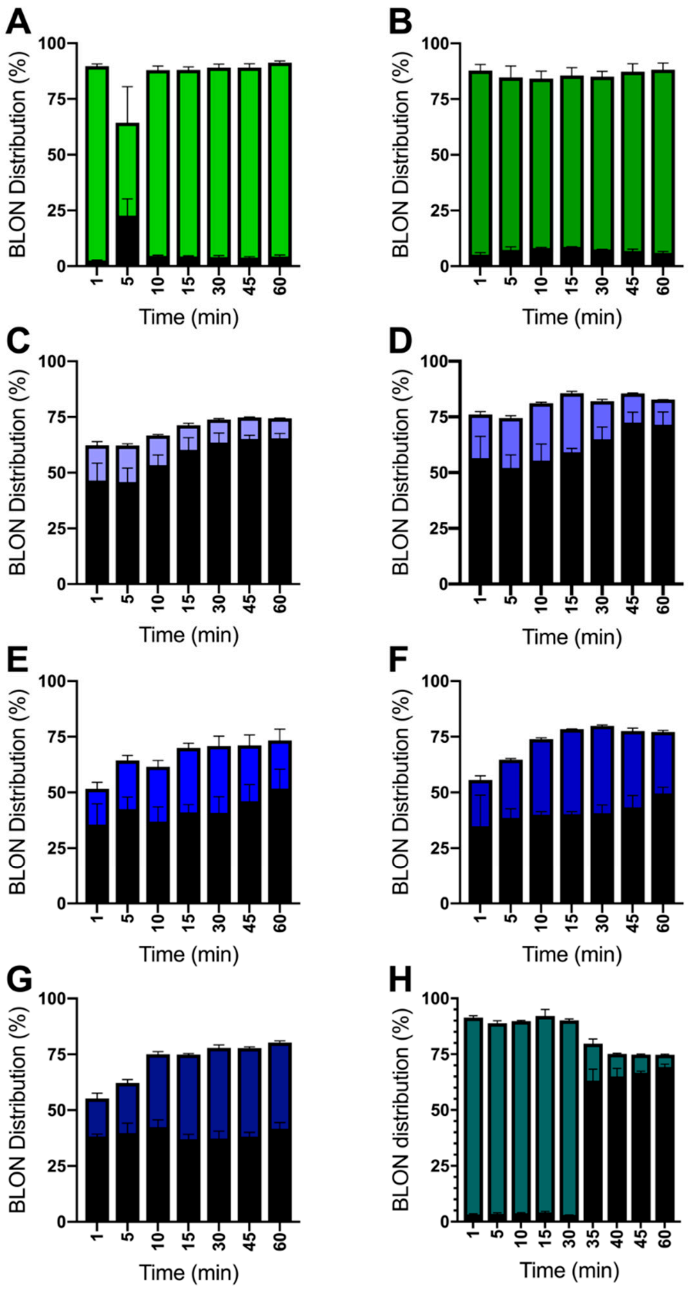

Figure S6: BLON distribution between aqueous (coloured) and pellet phase (black) during *in vitro* lipolysis (pH 6.5). A) liquid super-SNEDDS150%, B) liquid super-SNEDDS250%, C) solid super-SNEDDS90%, D) solid super-SNEDDS150%, E) solid super-SNEDDS200%, F) solid super-SNEDDS250%, G) solid super-SNEDDS300% and H) liquid super-SNEDDS with addition of silica microparticles after 30 min. Each value represents the mean ± SD, n = 3. Data for liquid super-SNEDDS150% and solid

super-SNEDDS150% was previously reported by and is published with permission from (Møller et al., 2021).

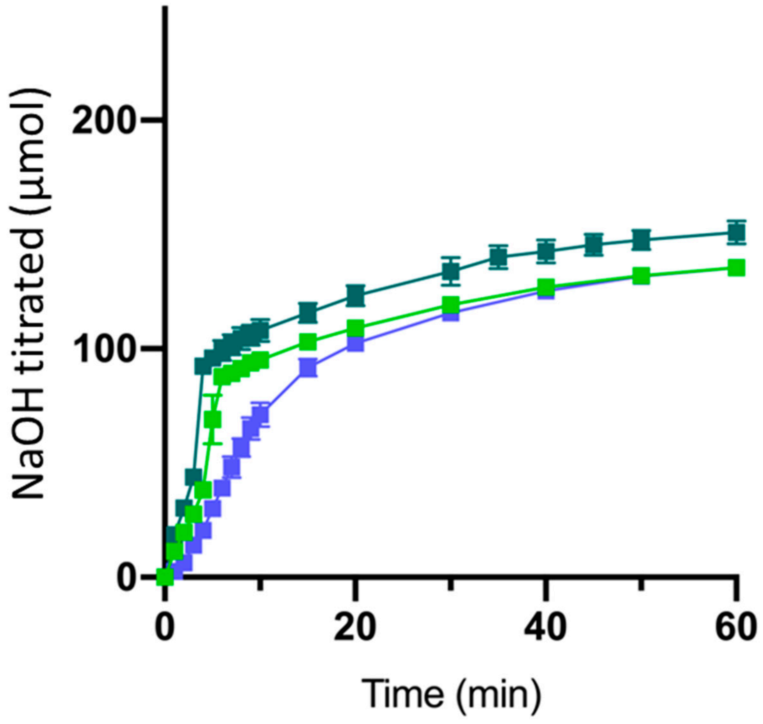

Figure S7: The digestion profiles during *in vitro* lipolysis (pH 6.5) of liquid super-SNEDDS150% (square, light green), solid super-SNEDDS150% (blue, square) and liquid super-SNEDDS150% with addition of silica after 30 min (teal, square). Each value represents the mean ± SD, n = 3. Data for formulation liquid super-SNEDDS150% and solid super-SNEDDS150% were previously reported by and is published with permission from (Møller et al., 2021).

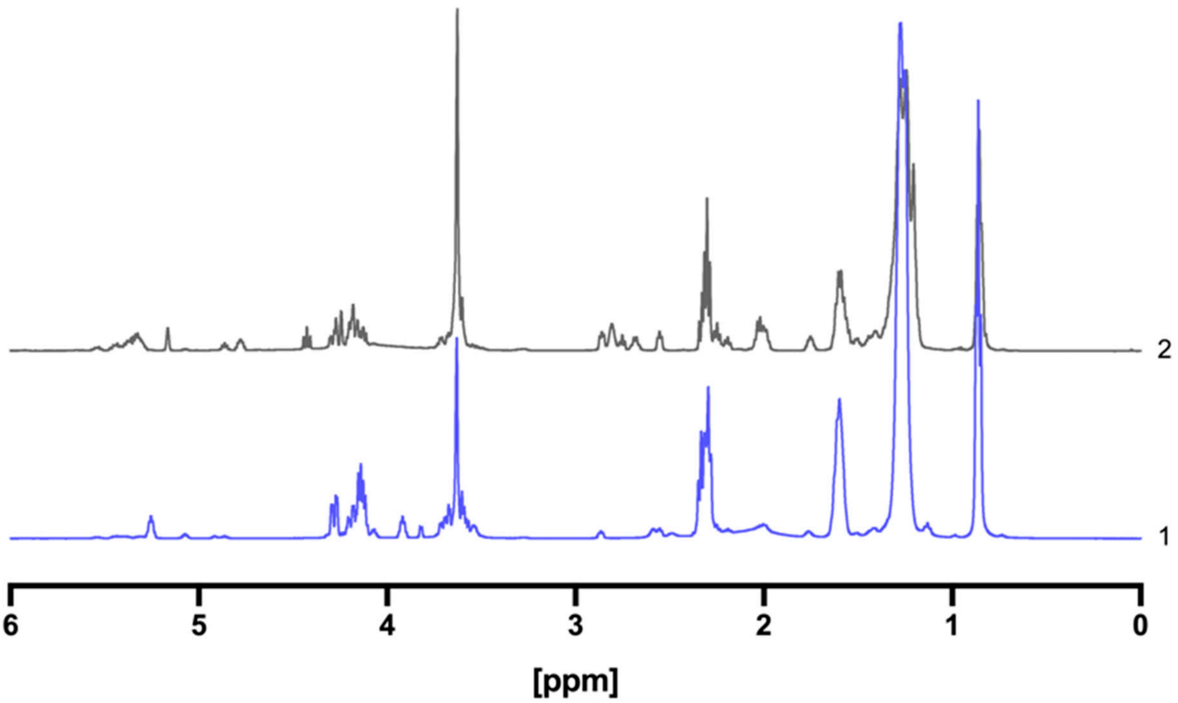

Figure S8: NMR spectra of 1) undigested solid super-SNEDDS150% and 2) solid super-SNEDDS150% after 60 min of lipolysis.

References

Møller, A., Schultz, H.B., Meola, T.R., Müllertz, A., Prestidge, C.A., 2021. The Influence of Solidification on the in vitro Solubilisation of Blonanserin Loaded Supersaturated Lipid-Based Oral Formulations. European Journal of Pharmaceutical Sciences 157, 105640.
